# A case of bilateral self-induced keratoconus in a patient with tourette syndrome associated with compulsive eye rubbing: case report

**DOI:** 10.1186/1471-2415-11-28

**Published:** 2011-09-21

**Authors:** Artemios Kandarakis, Michael Karampelas, Vasileios Soumplis, Christos Panos, Nikolaos Makris, Stylianos Kandarakis, Dimitrios Karagiannis

**Affiliations:** 1 1st Ophthalmology Department, Ophthalmiatrion Eye Hospital of Athens, Sina 2, Athens, 106 72, Greece; 2 Moorfields Eye Hospital, 162 City Road,London ,EC1V 2PD, UK; 3 Ophthalmology Department, Weill Cornell Medical College, 1305 York Avenue at E. 70th Street, New York, NY 10021, USA

## Abstract

**Background:**

Tourette syndrome is a neurologic disorder that is characterized by repetitive muscle contractions that produce stereotyped movements or sounds. Approximately 50% of individuals with TS also exhibit obsessive-compulsive behaviors including eye rubbing. We report a case of bilateral self-induced keratoconus in a patient with TS, associated with compulsive eye rubbing.

**Case presentation:**

A 35-year-old man was first seen in our clinic as an outpatient due to rapid deterioration of vision in his right eye associated with pain and tearing, over a period of one month. Slit lamp biomicroscopy of the right eye showed a central stromal scar due to corneal hydrops. Clinical examination and corneal topography of the left eye were normal. Six months later the patient developed corneal hydrops of his left eye. During the following examinations his vision continued to deteriorate in both eyes, while a central stromal scar was forming in his left cornea. Four years after the initial examination the patient's visual acuity was no light perception in the right eye and counting fingers at 33 cm in the left eye. His right eye was phthisic.

**Conclusions:**

Our patient developed a rapidly progressing bilateral corneal ectasia and phthisis of his right eye during a time period of 4 years. This unusual pattern suggests that the patient's compulsive behavior compromised both of his corneas and led to bilateral keratoconus.

## Background

Tourette syndrome (TS) was first described in 1885 by French physician Gilles de la Tourette. TS is a neurologic disorder that is characterized by repetitive muscle contractions that produce stereotyped movements (motor tics) or sounds (vocal tics). Approximately 50% of individuals with TS also exhibit obsessive-compulsive behaviors [1]. Eye tics (blinking, alterations in gaze, rubbing of the eyes, blepharospasm, etc.) are well known as early manifestations of TS [2], while there are reports which associate this syndrome with color vision deficiencies [3], self-induced bilateral retinal detachment [4] and visual field defects [5]. We report a case of bilateral self-induced keratoconus in a patient with TS, associated with compulsive eye rubbing. To the best of our knowledge this is the first longitudinal report of corneal ectasia associated with TS, describing the progression of keratoconus during a follow-up period of four years. This case report follows all the guidelines required by the Ethics Committee of which all authors are affiliated.

## Case Presentation

A 35-year-old man (K.E) was first seen in our clinic as an outpatient due to a rapid deterioration of vision in his right eye associated with pain and tearing, over a period of one month. He also noted a gradual decline of vision in his right eye over the previous year. His medical history included TS diagnosis since childhood, asthma, mild depression and low grade myopia in both eyes from the age of 7. He was on aripiprazol, clonazepam and sertraline. During the examination the patient did not have extensive vocal tics but he manifested several motor tics including frequent bilateral eye rubbing. The patient did not have any symptoms of ocular itching or irritation and on questioning he reported vigorous rubbing in both eyes.

**Figure 1:**
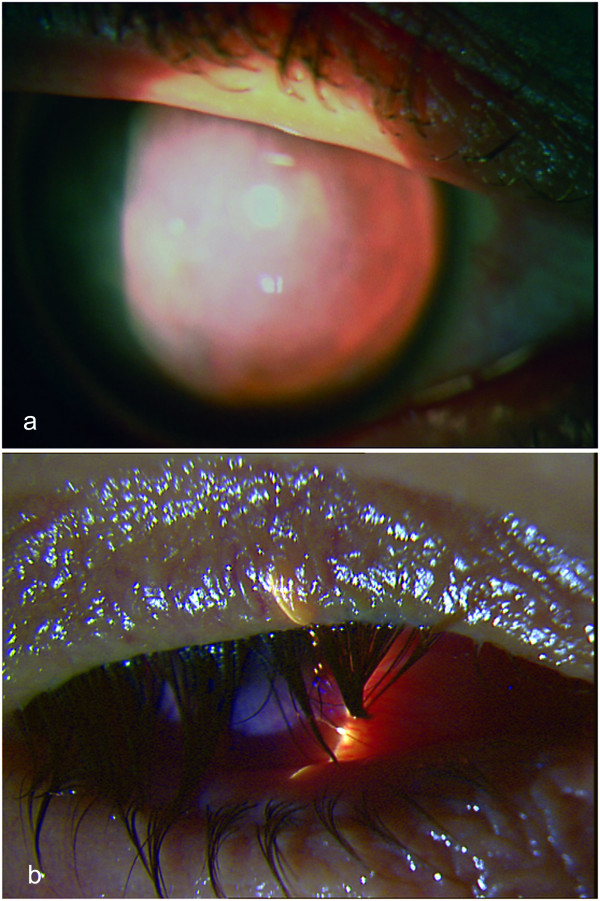
**Biomicroscopic appearance of the right eye at presentation showing central stromal scar (a) and 4 years after presentation showing phthisis (b)**. Short title: Biomicroscopic appearance of the right eye at presentation and 4 years later.

**Figure 2:**
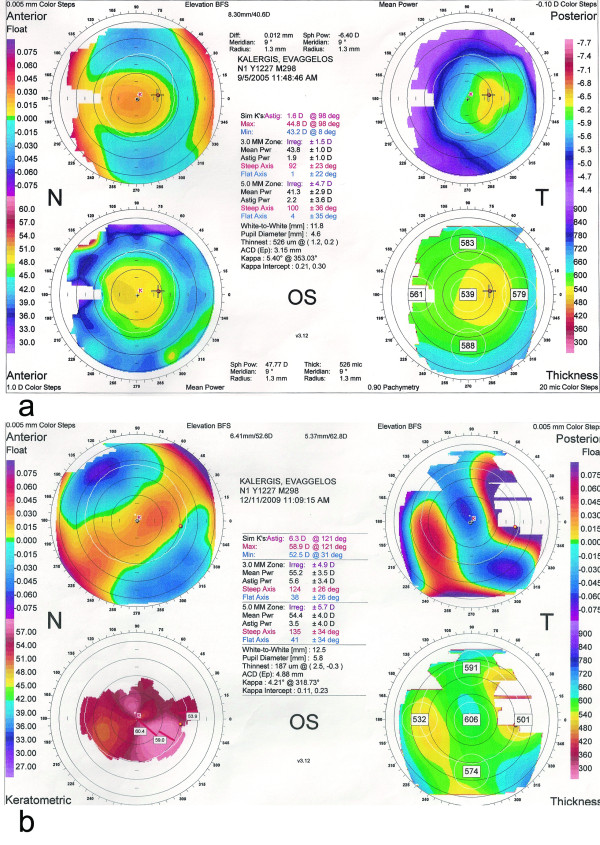
**Corneal topography of the left eye at presentation with normal findings (a) and 4 years after presentation showing keratoconus (b)**. Short title: Corneal topography of the left eye at presentation and 4 years later.

At baseline examination, best corrected visual acuity (BCVA) was counting fingers at 0,5 m in the right eye and 9/10 in the left eye (with -2,50 sph, -1,50 cyl × 90 correction). Slit lamp biomicroscopy of the right eye showed a central stromal scar secondary to corneal hydrops (Figure 1a), while his left eye was normal. There was no evidence of papillary or follicular lesions on both tarsal conjunctivae. His intraocular pressures were normal. Corneal topography of his left eye was normal (Figure 2a).

Axial lengths as measured by immersion A-scan ultrasonography were 25,14 mm in the right eye and 24,71 mm in the left eye, while B-scan ultrasonography did not show any pathologic findings.

We treated the right eye with hypertonic saline drops, cycloplegic drops and advised the patient to avoid eye rubbing.

Six months later, the patient presented complaining of sudden drop in vision in his left eye. BCVA was counting fingers at 33 cm in his right eye and had declined to 1/10 in the left eye. Slit lamp biomicroscopy showed corneal hydrops of the left eye with accompanying epithelial loss. We treated the left eye with topical hypertonic saline drops, cycloplegic drops, oral acetazolamide, an eye shield at night and strongly advised him to avoid eye rubbing. We also contacted the patient's general practitioner which informed us that the patient did not had a close psychiatric surveillance, while he failed to appear in many of his routine appointments over the last years.

**Figure 3:**
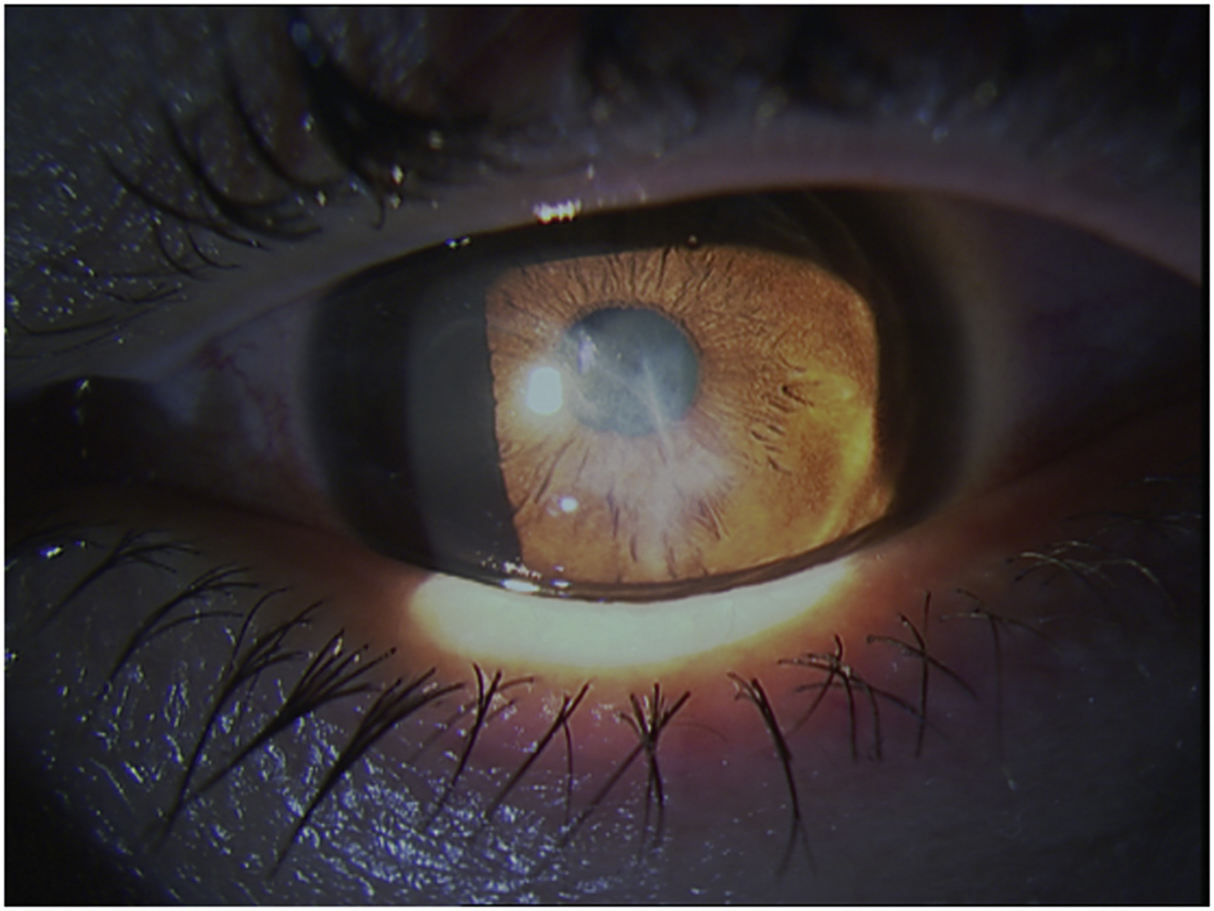
**Biomicroscopic appearance of the left eye showing central stromal scar**.

One year later BCVA was hand motion in his right eye and counting fingers at 2 m in his left eye with normal light projections while during the following months his vision continued to deteriorate in both eyes with central stromal scar formation in the left cornea (Figure 3). Corneal topographies of his left eye showed a corresponding progression (Figure 2b). Four years after presentation BCVA was no light perception in the right eye and counting fingers at 33 cm in the left eye. His right eye was phthisic (Figure 1b).

Keratoconus is a bilateral non-inflammatory corneal ectasia with an incidence of approximately 1 per 2000 in the general population [6]. Although its most common presentation is as an isolated sporadic disorder, it has also been associated with various diseases. An indication of a connection between keratoconus and TS is implied in a preliminary report by Enoch et al [5]. In this report which primarily investigates visual field defects in a group of 12 patients with TS, it is noted that two patients exhibited keratoconus, with no further analysis concerning the aetiologic relationship between the two conditions. Furthermore, Mashor et al recently described three cases of patients with TS-provoked eye rubbing that exhibited keratoconus [7].

There are multiple studies that report a high association of eye rubbing with keratoconus, but a cause-and-effect relationship is difficult to be established [8]. The association is implied by the high percentage of keratoconus patients with a positive history of eye rubbing. The reported prevalence ranges from 66% to 73% [9]. Rabinowitz performed a case-control study which showed that keratoconus patients do rub their eyes more often than normal controls (80% versus 58%, p < 0.001) [6]. Furthermore, keratoconus relates to a variety of conditions in which eye rubbing is a common feature like vernal atopic disease [10], Trisomy 21 [11] and Leber's tapeto-retinal degeneration [12]. Mechanical trauma has been implicated in the pathogenesis of keratoconus and the proposed mechanisms include increased apoptosis and increased oxidative damage [13].

## Conclusions

Our patient developed a rapidly progressing bilateral corneal ectasia and phthisis of his right eye during a time period of 4 years. It must be emphasized that although his left eye was normal at the initial examination, it developed corneal hydrops in a period of six months. This unusual pattern indicates that the patient's compulsive behavior compromised both of his corneas and led to bilateral keratoconus.

In conclusion, TS can lead to severe keratoconus associated with compulsive eye rubbing. It is therefore essential to assist patients with TS that exhibit this kind of behavior by suggesting the use of protective polycarbonate goggles and by ensuring a close surveillance in conjunction with the psychiatry service.

## Consent

Written informed consent was obtained from the patient for publication of this case report and any accompanying images.

## Competing interests

The authors declare that they have no competing interests.

## Authors' contributions

AK, MK, VS, CP, NM, SK and DK treated the patient and in doing so acquired the case data; they were also involved with drafting of the manuscript. All authors read and approved the final manuscript.

## Pre-publication history

The pre-publication history for this paper can be accessed here:

http://www.biomedcentral.com/1471-2415/11/28/prepub
